# Overexpression of angiotensin-converting enzyme 2 by renin-angiotensin system inhibitors. Truth or myth? A systematic review of animal studies

**DOI:** 10.1038/s41440-021-00641-1

**Published:** 2021-03-10

**Authors:** Hisashi Kai, Mamiko Kai, Hiroshi Niiyama, Norihito Okina, Motoki Sasaki, Takanobu Maeda, Atsushi Katoh

**Affiliations:** 1 Department of Cardiology, Kurume University Medical Center, Kurume, Japan; 2 Department of Pharmaceutical and Health Care Management, Faculty of Pharmaceutical Sciences, Fukuoka University, Fukuoka, Japan

**Keywords:** Angiotensin receptor blockers, Angiotensin-converting enzyme inhibitors, mRNA expression, Protein expression, COVID-19

## Abstract

Angiotensin-converting enzyme 2 (ACE2) protects against organ damage in hypertension and cardiovascular diseases by counter regulating the renin-angiotensin system (RAS). ACE2 is also the receptor for severe acute respiratory syndrome coronavirus 2 (SARS-CoV-2). Based on the claim that RAS inhibitors (RASIs) cause ACE2 overexpression in some animal experiments, concerns have arisen that RASIs may aggravate SARS-CoV-2 infection and coronavirus disease-2019 severity in RASI-treated patients. To achieve a comprehensive review, a systematic search of MEDLINE/PubMed was conducted regarding the effects of RASIs on tissue ACE2 mRNA/protein expression in healthy animals and animal models of human diseases. We identified 88 eligible articles involving 168 experiments in the heart, kidneys, lungs, and other organs. Three of 38 experiments involving healthy animals showed ACE2 expression greater than twice that of the control (overexpression). Among 102 disease models (130 experiments), baseline ACE2 was overexpressed in 16 models (18 experiments) and less than half the control level (repression) in 28 models (40 experiments). In 72 experiments, RASIs did not change ACE2 levels from the baseline levels of disease models. RASIs caused ACE2 overexpression compared to control levels in seven experiments, some of which were unsupported by other experiments under similar conditions. In 36 experiments, RASIs reversed or prevented disease-induced ACE2 repression, yielding no or marginal changes. Therefore, ACE2 overexpression appears to be a rare rather than common consequence of RASI treatment in healthy animals and disease models. Future studies should clarify the pathophysiological significance of RASI-induced reversal or prevention of ACE2 repression in disease models.

## Introduction

Angiotensin-converting enzyme 2 (ACE2) is a membrane-bound carboxydipeptidase that converts angiotensin (Ang) I into Ang1-9 and AngII into Ang1-7 [1, 2]. In turn, Ang1-7 activates the mas receptor, which inhibits the physiological and pathophysiological actions of AngII type 1 receptor (AT1R) activation. ACE2 is therefore considered an intrinsic counter-regulator of the renin-angiotensin system (RAS) and antagonizes RAS-mediated deleterious effects in not only cardiovascular disease (CVD) but also acute lung injury [3, 4].

Emphasis has been placed on the results of some animal experiments reporting that the administration of AT1R blockers (ARBs) and angiotensin-converting enzyme inhibitors (ACEIs) led to tissue ACE2 overexpression in the cardiovascular system [2, 5–9]. This is truly at the crux of a current problem and its associated confusion, in that ACE2 has also been identified as the receptor for severe acute respiratory syndrome coronavirus 2 (SARS-CoV-2), the virus responsible for coronavirus disease 2019 (COVID-19) [10–12]. This finding raised concerns that ARBs and ACEIs may augment susceptibility to SARS-CoV-2 infection and aggravate the severity of COVID-19 in hypertensive or CVD patients receiving these drugs [7, 8]. However, discussions on this issue have been based on literature reviews that have included only a limited number of articles, focused on those involving the heart and kidneys. In addition, many studies have shown that tissue ACE2 is downregulated in a variety of animal disease models [3, 4]. Nevertheless, several studies of ACE2 expression have conducted comparisons only between animals with and without drug administration and have lacked comparisons with adequate control or sham groups [13–15]. Similarly, previous reviews have tended to focus on RAS inhibitor (RASI)-induced ACE2 expression changes compared to the baseline levels of disease models [2, 5–8]. Such comparisons may lead to overestimation of RASI-induced ACE2 overexpression beyond the levels in healthy or control animals.

In this study, to perform a comprehensive review, we adopted the search strategy and predetermined population, intervention, comparison, and outcome (PICO) framework for systematic review recommended by the Preferred Reporting Items for a Systematic Review and Meta-analysis (PRISMA) statement [16]. This method has recently been applied to pathological investigations, although it was originally intended for clinical research [17]. In addition, the relative changes in the baseline and drug-treated ACE2 levels compared to the adequate control levels were investigated in animal models of human diseases.

## Methods

### Search strategy

We conducted the current comprehensive review by applying a search strategy for systematic review according to the PRISMA statement [16]. A modified PICO framework was used to structure the review process [16] and adapted to experimental research studies involving animal tissues. Participants were healthy animals or animal models of human diseases. Intervention was chronic treatment with RASIs. Competitors were control or sham animals, that is, vehicle-treated healthy animals or normal (i.e., normotensive or normoglycemic), sham-operated or wild-type control animals of the disease models. Outcomes were the relative changes in the tissue expression of ACE2 mRNA or protein compared to control or sham.

We conducted a systematic search of MEDLINE/PubMed from January 1965 to June 2020 and updated the search on August 31, 2020. The search equations used for the systematic search are shown in Supplementary Table 1. The publication type was limited to original investigations in adult animals. We excluded ex vivo experiments and short-term studies using observation periods within 3 days.

### Study screening

After the database search, the retrieved articles were subjected to the first screening step by two independent authors (HN and AK) to remove duplicates and irrelevant studies based on the titles and abstracts. Thereafter, assessment of the full-text articles for eligibility was performed as the second screening step.

### Data extraction and evaluation

Data on mRNA and protein expression were extracted from the text, tables, and figures of each article by three independent authors (NO, MS, and TM). Fold-changes in ACE2 expression levels were obtained by comparing the RASI-treated group to the control or sham group. In experiments examining both mRNA and protein expression, mRNA data were used for subsequent analyses if the degree of ACE2 mRNA expression change was greater than that of the protein expression change, and vice versa. Expression changes were semiquantitatively graded as follows: overexpression, when the ACE2 expression level was greater than twice that in the control group; repression, when the ACE2 expression level was less than half that in the control group; and no/marginal change, when the ACE2 expression remained at a level from half to twice that of the control group.

## Results

### Studies included

The database search extracted 346 articles that were subjected to the first screening step (Supplementary Fig. 1). After the second screening step, 88 articles (168 experiments) remained. Among these, 65 articles investigated ARBs, 30 investigated ACEIs, 5 investigated MRAs, 3 investigated aliskiren, 1 investigated sacubitril/valsartan, and 1 investigated hydrochlorothiazide (Supplementary Tables 2–4).

### Healthy normal animals

**Fig. 1:**
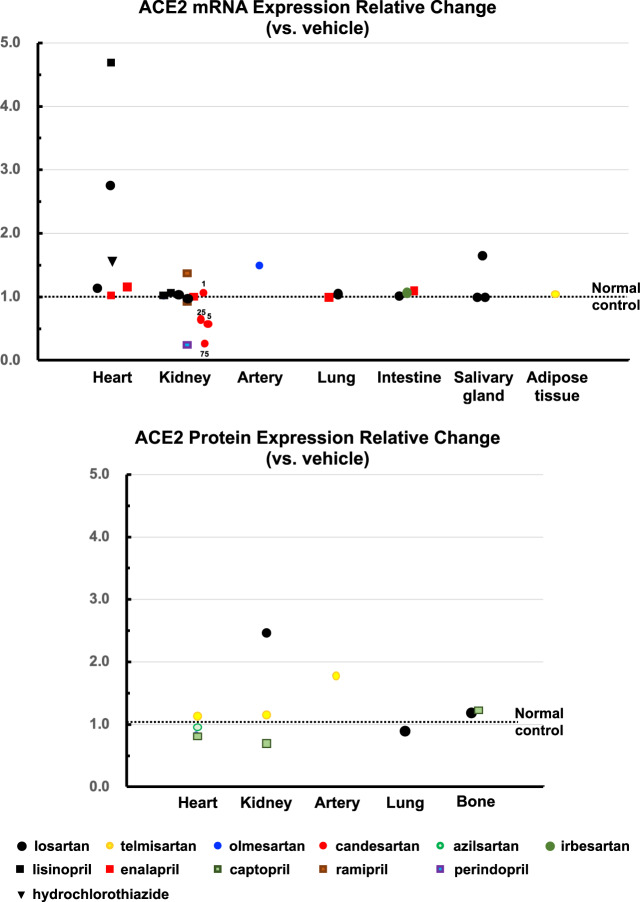
Effects of RASIs on ACE2 expression in healthy normal animals

In healthy normal animals, the effects of RASIs on ACE2 mRNA or protein expression were investigated in 21 articles, including 26 models and 38 experiments (Fig. 1). Among these, RASIs induced ACE2 overexpression in three experiments involving the heart or kidneys and ACE2 repression in two experiments involving the kidneys. However, the results were not necessarily supported by other experiments under similar conditions. The remaining 33 experiments demonstrated that ARBs, ACEIs, and hydrochlorothiazide did not change ACE2 expression in the heart, kidneys, arteries, lungs, intestine, or other organs.

### Heart

Losartan induced cardiac ACE2 mRNA overexpression in Lewis rats [18] but did not change ACE2 mRNA expression in C57BL/6 mice (Fig. 1) [19]. Azilsartan did not alter cardiac ACE2 protein levels in db/+ mice [20]. Cardiac ACE2 mRNA was unchanged by enalapril in C57BL/6 mice or Sprague-Dawley (SD) rats [19, 21] but was overexpressed by lisinopril in Lewis rats [18]. Cardiac ACE2 protein was unaffected by captopril in Wistar rats [22] and C57BLKS/J mice [23]. Hydrochlorothiazide did not change cardiac ACE2 mRNA levels in Wistar rats [24].

### Kidneys

ACE2 mRNA in the whole kidney or renal cortex was unchanged by losartan in Lewis rats and C57BL/6 mice [19, 25], by candesartan at 1, 5, and 25 mg/kg/day in db/+ mice [26], by lisinopril in Lewis rats or Wistar Kyoto rats (WKY) [25, 27], by enalapril in C57BL/6 mice [19], or by captopril in C57BLKS/J mice [23]. Ramipril did not change ACE2 mRNA levels in either the renal cortex or medulla of SD rats [28]. Renal ACE2 protein was unaffected by telmisartan or captopril in C57BLKS/J mice [23]. In C57BL/6 mice, losartan induced overexpression of renal ACE2 protein [29], whereas perindopril repressed renal ACE mRNA [30]. Although candesartan at 75 mg/kg/day repressed renal ACE2 protein in db/+ mice [26], this dose was too high to be regarded as having a therapeutic or pharmacological effect.

### Arteries

Olmesartan did not change ACE2 mRNA levels in the femoral artery of C57BL/6 mice [31]. A superselective preparation technique using confocal microscopy and laser capture microdissection revealed that telmisartan did not change mRNA in renal arterioles of C57BL/6 mice [32].

### Lungs

Losartan and enalapril did not change pulmonary ACE2 mRNA in C57BL/6 mice [19]. Losartan did not affect pulmonary ACE2 protein in SD rats [33].

### Gastrointestinal tract

In the intestine, ACE2 mRNA was unchanged by losartan, irbesartan, or enalapril in C57BL/6 mice [19, 34]. In Wistar rats, losartan did not change ACE2 mRNA in the parotid, submandibular, or sublingual glands [35].

### Other tissues

Telmisartan had no effect on ACE2 mRNA in the peritoneal adipose tissue of Wistar rats [36]. Losartan and captopril did not change ACE2 protein levels in the femoral head in Wistar rats [37, 38].

### Animal models of human diseases

In 130 experiments involving 102 animal models of human diseases, baseline ACE2 was overexpressed in 16 models (18 experiments) and repressed in 28 models (40 experiments) (Table 1). The remaining 58 models (72 experiments) demonstrated that ACE2 expression remained within the no/marginal change level. RASIs increased ACE2 from the control to overexpression levels in seven experiments, some of which were unsupported by other experiments under similar conditions. In 36 experiments, disease-induced ACE2 changes were reversed or prevented by RASIs to yield no/marginal change. In 74 experiments, RASIs did not affect the ACE2 level in disease models.

**Table 1:** Changes in ACE2 expression levels induced by renin-angiotensin system inhibitors in animal models of human diseases

The number of experiments	Under treatment			
	Overexpression	No/marginal change	Repression	Sum
Baseline				
Overexpression	12	5	1	18
No/marginal change	7	58	7	72
Repression	0	36	4	40
Sum	19	99	12	130

Overexpression was defined when ACE2 expression was greater than twice as much as the control group. Repression was defined when ACE2 expression was less than half of the control group. No/marginal change was defined when ACE2 expression remained within 1/2 to 2 times of the control group

*ACE2* angiotensin-converting enzyme 2

### Heart disease models

#### Hypertension

**Fig. 2:**
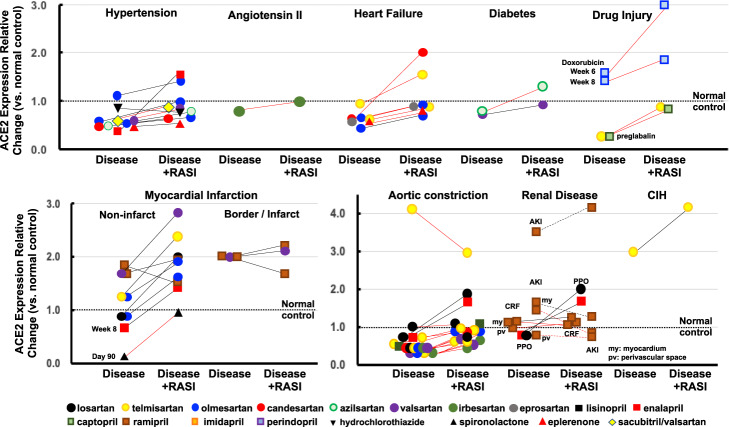
Effects of RASIs on ACE2 expression in heart disease models. Changes in mRNA and protein expression are represented as black and red lines, respectively

In spontaneously hypertensive rats (SHRs), cardiac ACE2 protein levels showed no/marginal change in two articles [24, 39], whereas cardiac ACE2 mRNA was repressed in another article (Fig. 2) [40]. Regarding other hypertensive models, cardiac ACE2 was unchanged at the mRNA level in stroke-prone SHRs (SHR-SP), Ren2-transgenic (tg) mice, or hRN/hANG-tg mice [41–43] and at the protein level in AngII-infused C57BL/6 mice [44]. In these experiments, cardiac ACE2 mRNA was unaffected by olmesartan in SHR-SP, Ren-tg mice, and hRN/hANG-tg mice [41–43], by azilsartan in hRN/hANG-tg mice [43], and by HCTZ in SHRs [24]. ACE2 protein was also unchanged by valsartan or sacubitril/valsartan in SHRs or by irbesartan in AngII-infused C57BL/6 mice [39, 44]. One article showed that enalapril restored cardiac mRNA from a repression level to the no/marginal change level [40]. Two other hypertensive models reported cardiac ACE2 repression: in Dahl salt-sensitive rats, cardiac ACE2 mRNA was barely reduced to repression level and reversed to the no/marginal change level by candesartan and eplerenone [45], and in N^G^-L-arginine methyl ester (LNAME)-treated hRN/hANG-tg mice, cardiac ACE2 mRNA was repressed and restored by olmesartan to the no/marginal change level [46].

#### Pressure overload

In an article comparing ARBs, thoracic aortic constriction (TAC) repressed cardiac ACE2 protein in C57BL/6 mice at weeks 2 and 4 [47]. This TAC-induced ACE2 repression was prevented at the no/marginal change level by candesartan, losartan, olmesartan, irbesartan, telmisartan, or valsartan in the week 2-model and by olmesartan, telmisartan, or valsartan in the week 4-model [47]. In another experiment in SD rats receiving TAC, cardiac ACE2 protein levels showed no/marginal change (but nearly at repression level) at week 8, which was unchanged by telmisartan [48].

In Wistar rats, abdominal aortic constriction (AAC) reduced cardiac ACE2 protein to barely repression level at week 3, which was prevented at the no/marginal change level by captopril [22]. On immunohistochemistry, AAC did not change ACE2 immunointensity in coronary arteries at week 3, which was unaffected by losartan [49]. In SD rats at 4 weeks after AAC, cardiac ACE2 mRNA remained at the no/marginal change level and was unaffected by losartan or enalapril [50]. Further, AAC had no effect on ACE2 immunointensity in the myocardial interstitium or vessels at week 16, which was unaffected by telmisartan [51]. In contrast, in SD rats with ACC associated with reduced left ventricular ejection fraction at week 5, cardiac ACE2 protein was overexpressed, with slight attenuation still within overexpression levels by telmisartan [52].

#### Heart failure

In preventive experiments of heart failure with reduced ejection fraction (HFrEF) due to experimental autoimmune myocarditis (EAM), 3 weeks of treatment with an ARB was started simultaneously with myosin immunization. Cardiac ACE2 protein was reduced to barely repressed levels, and this was prevented by olmesartan in one experiment [53]. In addition, cardiac ACE2 levels remained within the no/marginal change level in both models with or without telmisartan treatment [54]. In therapeutic experiments, an ARB was started at week 4 when HFrEF had been established. At week 8, EAM repressed cardiac ACE2 mRNA, which was reversed to the no/marginal change level by olmesartan [55]. At week 8, cardiac ACE2 protein remained within the no/marginal change level and was barely increased to the overexpression level by candesartan but not by telmisartan [56, 57].

In Wistar rats receiving aorto-caval fistula as a model of high cardiac output heart failure, cardiac ACE2 protein was within the no/marginal change level and unaffected by eprosartan or spironolactone [58]. In a broiler chicken model of pulmonary hypertension (PH), low temperature did not change ACE2 mRNA in the right ventricle, which was unaffected by imidapril [59].

#### Myocardial infarction

In the infarct/border myocardium of a myocardial infarction (MI) model at week 4, ACE2 mRNA was increased to barely overexpression level [60, 61]. Valsartan did not affect ACE2 overexpression [60]. Ramipril reduced the elevated ACE2 to the no/marginal change level in one experiment [61] but did not result in any change in another experiment [60]. In the viable myocardium of an MI model at week 4, ACE2 mRNA remained within the no/marginal change level in all six experiments [60–63]. Losartan, olmesartan, or ramipril resulted in unchanged ACE2 mRNA levels in the viable myocardium [60–63]. Telmisartan or valsartan increased the viable myocardial ACE2 mRNA from the no/marginal change level to the overexpression level [60, 63].

In the long-term MI models, ACE2 mRNA levels in the viable myocardium were within the no/marginal change level at week 8, which was not affected by enalapril [21]. In the border/infarct myocardium at day 90, ACE2 protein was repressed, with reversal to the no/marginal change level by spironolactone [64].

#### Diabetic cardiomyopathy

Cardiac ACE2 mRNA in streptozotocin (STZ)-induced diabetic mice and the ACE2 protein in db/db mice were within the no/marginal change level, and these results were unaffected by valsartan and azilsartan, respectively [20, 65].

#### Cardiorenal syndrome

In SD rats 10 days after subtotal nephrectomy (STNx), a model of acute kidney injury (AKI), cardiac ACE2 mRNA was within the no/marginal change level and unaffected by ramipril [66]. However, in another experiment, cardiac ACE2 mRNA was overexpressed, but ACE2 immunointensity was within the no/marginal change level in the myocardium or perivascular space [67]. Ramipril did not affect cardiac ACE2 mRNA and ACE2 immunointensity in the myocardium or perivascular space. In SD rats at 4 weeks after STNx, a model of chronic renal failure (CRF), cardiac ACE2 mRNA was within the no/marginal change level and was unaffected by ramipril [68]. Immunohistochemistry confirmed that the ACE2 immunointensity in the myocardium or perivascular space was unchanged with and without ramipril treatment [68]. In mice with hydronephrosis induced by unilateral ureter occlusion (UUO), cardiac ACE2 mRNA was within the no/marginal change level and was overexpressed with losartan but not with enalapril [69].

#### Drug-induced cardiomyopathy

In Wistar rats, doxorubicin did not change cardiac ACE2 protein levels at weeks 6 and 8 [70]. When cilazapril was started at week 4, cardiac ACE2 protein was overexpressed at week 6, returning to the no/marginal change level at week 8. Pregabalin had no effect on cardiac ACE2 protein at the no/marginal change level, which was unaffected by captopril or telmisartan [71].

#### Sleep apnea syndrome

In mice receiving chronic intermittent hypoxia as a model of sleep apnea syndrome (SAS), cardiac ACE2 mRNA was overexpressed and was further augmented by telmisartan [72].

### Kidney disease models

#### Hypertension

**Fig. 3:**
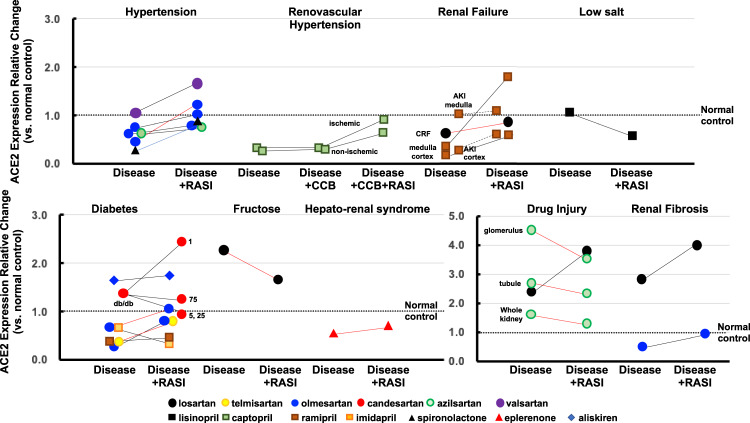
Effects of RASIs on ACE2 expression in kidney disease models. Black lines, mRNA changes; red lines, protein changes

Renal ACE2 mRNA was repressed in ischemic and nonischemic kidneys in the 2-kidney 1-clip model and in heminephrectomized SD rats receiving aldosterone and a high salt diet, with reversal to the no/marginal change level by captopril and spironolactone, respectively (Fig. 3) [73, 74]. In SHR-SP, renal ACE2 protein was repressed and restored to the no/marginal change level by olmesartan [41]. Renal mRNA showed no/marginal change in Ren2-tg mice and hRN/hANG-tg mice [43, 75, 76]. Valsartan or olmesartan did not affect ACE2 expression in Ren2 mice [75, 76], and olmesartan or azilsartan showed no effect in hRN/hANG-tg mice [43].

#### Diabetic nephropathy

In SD rats, STZ repressed renal ACE2 mRNA, which was reversed to the no/marginal change level by olmesartan [77]. STZ also repressed renal tubular ACE2 mRNA in SD rats, which was unchanged by ramipril [78]. In C57BL/6 mice, STZ repressed renal ACE2 protein, which was reversed to the no/marginal change level by telmisartan in one experiment [79], while another experiment revealed renal ACE2 mRNA within the no/marginal change level, showing no effect of olmesartan [80]. In STZ-treated C57BL/6 mice, nonobese diabetic (NOD) mice, and db/db mice, renal ACE2 mRNA was unchanged at the no/marginal change level [26, 30, 81]. Perindopril repressed renal ACE2 mRNA in STZ-treated C57BL/6 mice [30]. Aliskiren did not change renal mRNA at the no/marginal change level in NOD mice [81]. In db/db mice, although a low dose of candesartan (1 mg/kg/day) promoted overexpression of renal ACE2 mRNA, higher doses of candesartan (5, 25, 75 mg/kg/day) did not change the expression from the no/marginal change level [26].

#### Renal failure

In an AKI model of SD rats, ACE2 mRNA was repressed in the renal cortex and restored to the no/marginal change level by ramipril, whereas medullary ACE2 was within the no/marginal change level and unaffected by ramipril [28]. Thus, ACE2 expression is suggested to be regulated differently in the cortex and medulla. In a CRF model of SD rats, ACE2 mRNA was repressed in the cortex and medulla, with reversal to the no/marginal change level by ramipril [68]. In the same CRF model, renal ACE2 protein was within the no/marginal change level, which was unaffected by losartan [82].

#### Renal fibrosis

In Col4a3-knockout mice, a model of Alport syndrome with renal fibrosis, renal ACE2 mRNA was repressed and restored to the no/marginal change level by olmesartan [83]. In C57BL/6 mice receiving UUO, another model of renal fibrosis, ACE2 mRNA was overexpressed in the contralateral kidney and further augmented by losartan [84].

#### Drug injury

In doxorubicin-treated BALB/c mice, a model of nephrotic syndrome, renal ACE2 mRNA was overexpressed and further augmented by losartan [85]. In adenine-induced CRF in WKY, ACE2 protein in the whole kidney was within the no/marginal change level and unaffected by azilsartan [86]. However, ACE2 immunointensity was increased to an overexpression level in the glomerulus or tubules, which was unchanged by azilsartan.

#### Others

In a mouse model of metabolic syndrome (MetS), a fructose-rich diet induced renal ACE2 protein overexpression, which was restored to the no/marginal change level by losartan [29]. In WKYs receiving a low-salt diet, renal ACE2 mRNA was within the no/marginal change level and was unaffected by lisinopril [27]. In SD rats with bile duct ligation (BDL), a model of hepatorenal syndrome, renal ACE2 protein was unchanged at the no/marginal change level and was unaffected by spironolactone [87].

### Vascular disease models

#### Hypertension

**Fig. 4:**
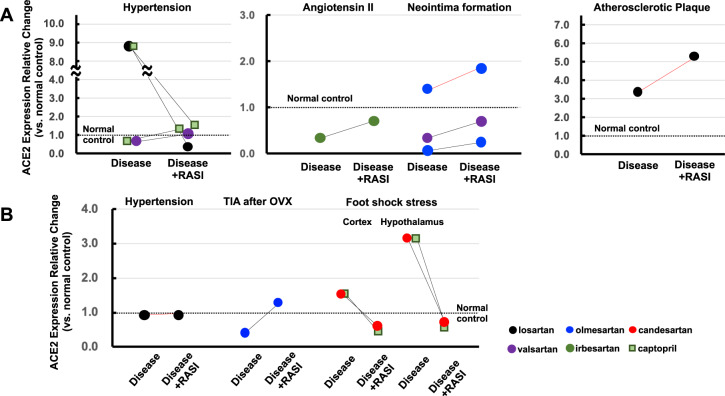
Effects of RASIs on ACE2 expression in artery (**A**) and brain (**B**) disease models. Black lines, mRNA changes; red lines, protein changes

In SHRs, one experiment showed that aortic ACE2 mRNA was within the no/marginal change level and was unaffected by captopril [88], whereas another revealed aortic mRNA overexpression, with reversal to the repression level by losartan or to the no/marginal change level by captopril (Fig. 4A) [89]. The reason for this discrepancy is unknown because the thoracic aorta was obtained from SHRs of the same age in weeks in both experiments. On the other hand, in SHR-SP, aortic ACE2 mRNA was within the no/marginal change level and was unaffected by valsartan [90]. In AngII-infused mice, aortic ACE2 mRNA was repressed, which was prevented by irbesartan at the no/marginal change level [91].

#### Neointima formation

Balloon injury repressed ACE2 mRNA in the thoracic aorta of Wistar rats, with reversal to the no/marginal change level by valsartan [92]. In SHRs, ACE2 protein levels in the carotid artery were within the no/marginal change level and were unaffected by olmesartan [93]. Cuff injury repressed ACE2 mRNA in the femoral artery of C56BL/6J mice, which was slightly attenuated to within the repression level by olmesartan [31].

#### Atherosclerotic plaque

In New Zealand white rabbits fed a high-cholesterol diet, ACE2 protein in the atherosclerotic plaque was overexpressed and further augmented by losartan [94].

### Brain disease models

In hRN/hANG-tg mice, brain ACE2 protein was within the no/marginal change level and unaffected by losartan (Fig. 4B) [95]. In SD rats of an ischemic stroke model subjected to middle cerebral artery occlusion, bilateral ovariectomy (OVx) repressed ACE2 mRNA in the peri-infarct area, which was prevented at the no/marginal change level by olmesartan [96]. In a stress model, foot shock stress overexpressed ACE2 mRNA in the hypothalamus but showed no change at the no/marginal change level in the cortex [97]. Candesartan and captopril prevented the ACE2 increase in the hypothalamus at the no/marginal change level. Captopril decreased cortical ACE2 from the no/marginal change to repression level, whereas candesartan resulted in no alteration at the no/marginal change level.

### Lung disease models

**Fig. 5:**
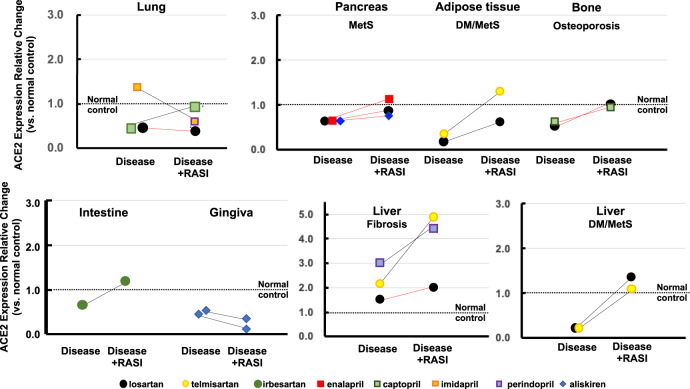
Effects of RASIs on ACE2 expression in disease models of various organs. Black lines, mRNA changes; red lines, protein changes

In a silicotic lung fibrosis model in Wistar rats, pulmonary ACE2 protein was repressed and restored to the no/marginal change level by captopril (Fig. 5) [98]. In Wistar rats, cigarette smoke-induced PH repressed pulmonary ACE2 protein, which was unchanged from the repression level by losartan [33]. In low temperature-exposed chickens, a PH model, pulmonary ACE2 mRNA was within the no/marginal change level and was unaffected by imidapril [99].

### Gastrointestinal tract/oral cavity disease model

In a mouse model of irritable bowel syndrome, stress did not change the intestinal ACE2 mRNA at the no/marginal change level and was unaffected by irbesartan (Fig. 5) [34]. In a periodontal disease model, gingival ACE2 mRNA was within the no/marginal change level in both nondiabetic and STZ-induced diabetic mice, both of which were repressed by aliskiren [100].

### Other disease models

#### Liver disease

In a mouse model of MetS, a high-fat diet repressed hepatic ACE2 mRNA, which was prevented at the no/marginal change level by losartan or telmisartan (Fig. 5) [101]. In SD rats receiving BDL, a rat model of liver fibrosis, hepatic ACE2 mRNA was within the no/marginal change level, which was increased by losartan to the barely overexpression level [102], whereas hepatic ACE2 protein was overexpressed and augmented further by telmisartan [103]. In another liver fibrosis model, CCl_4_ induced hepatic ACE2 mRNA overexpression, with perindopril inducing a further increase [104].

#### Pancreas

A high-fat diet did not change ACE2 protein in the pancreatic islets at the no/marginal change level, which was unaffected by enalapril, losartan, or aliskiren (Fig. 5) [105].

#### Adipose tissue

In STZ-induced diabetic rats fed a high-fat, high-sucrose diet, visceral fat ACE2 mRNA was repressed and slightly attenuated to within the repression level by losartan (Fig. 5) [106]. In subcutaneous fat, a high-fat diet repressed ACE2 mRNA, which was reversed to the no/marginal change level by telmisartan [36].

#### Osteoporosis

In the femoral head, OVx did not change the ACE2 protein, which was unaffected by losartan or captopril (Fig. 5) [37, 38].

## Discussion

Most articles included in this analysis aimed to investigate whether ACE2 is associated with the organ-protective mechanisms of RASIs. Among 58 of 102 disease models (72 and 130 experiments, respectively), RASIs did not affect ACE2 expression at baseline under disease conditions. Among 28 (40 experiments) of 102 disease models, ACE expression was repressed to less than half the control levels in the heart (hypertension, heart failure, and pressure-overload models), kidneys (hypertension, CRF, and diabetes models), arteries (AngII infusion and vascular injury models), lungs (silicosis model), and other organs (Table 1). Thirty-six of the 40 experiments showed that disease-induced ACE2 repression was prevented or restored by RASIs, yielding no/marginal change. Notably, no disease model showed that RASIs increased ACE2 expression from repression to overexpression levels. Many in vivo and in vitro studies have shown that ACE2 is a negative regulator of overactivated RAS and the resultant organ damage [1, 2, 4, 6]. In several diseased conditions, therefore, it is suggested that ACE2 is downregulated during organ damage and that prevention or restoration of ACE2 reduction may be involved in the organ-protective effects of RASIs. However, attenuation or prevention of ACE2 reduction may be secondary to the consequences of antihypertensive and/or the organ-protective effects of RASIs. This important issue should be addressed in future studies.

In the early days of ACE2 research, losartan and lisinopril were reported to cause overexpression of cardiac ACE2 mRNA in healthy rats [18]. Another study showed that losartan resulted in overexpression of renal ACE2 protein in healthy mice [29]. However, these findings were not supported by 7 additional experiments involving the heart and 14 experiments involving the kidneys (Fig. 1). [19–28, 30] In addition, RASIs did not affect ACE2 expression in the arteries, lungs, intestine, or other organs in healthy animals. [19, 31–38] With regard to animal models of human diseases, only 7 of the 130 experiments showed that RASIs increased ACE2 expression from control to overexpression levels in the heart, kidneys, and liver (Table 1) [26, 57, 60, 63, 69, 70, 102]. However, among these, 4 experiments of MI (viable myocardium), EAM, and diabetic nephropathy models [26, 57, 60, 63] were inconsistent with other experiments using different kinds or doses of ARBs or ACEIs in the same models (Figs. 2 and 3). [26, 55–57, 60–63] Whether these discrepancies are related to factors such as drug-specific effects, differences in the experimental conditions and/or the animal strains used should be determined. To clarify whether drug-specific effects exist, it is necessary to examine the effects of various kinds of ARBs or ACEIs in individual models.

Baseline ACE2 overexpression was observed in the heart of MI (infarct/border myocardium) and SAS models [60, 61, 72], in aortic atherosclerotic plaques [94], in the kidneys of AKI, drug injury, and UUO-induced fibrosis models, [67, 69, 84–86], and in the liver of BDL- or CCl4-induced fibrosis models [103, 104]. Interestingly, potent oxidative stress, fibroinflammatory changes, and mitochondrial damage are commonly found in such disease models. In addition, myocardial hypoxia and ischemia may also be related to ACE2 overexpression in the models of MI and SAS. The role of ACE2 overexpression in the pathogenesis of these disease models is a target of future research.

The doses used in most experiments were much higher than the therapeutic doses of RASIs. In addition, the doses of individual drugs used varied widely between experiments. Accordingly, the observed effects of RASIs cannot be extrapolated to humans. In addition, comparing the effects between drugs is difficult because the doses of each drug used were not optimized. In some articles, a small percentage of change in ACE2 expression was overemphasized as having statistical significance. Great care should be taken when interpreting such results because statistical significance does not necessarily translate directly to pharmacological or pathophysiological significance. In addition, methodological limitations exist in the detection and quantification of tissue mRNA and protein expression using currently available techniques, such as real-time polymerase chain reaction, Western blotting, and immunohistostaining. Moreover, many experiments studied relatively small numbers of animals and showed large variations in measured values, resulting in low and insufficient statistical power.

It is interesting to determine the effects of RASIs on ACE2 activity in healthy animals and animal models of human diseases. ACE2 activity was evaluated in several articles included in this study (Supplementary Tables 2–4). Although some studies showed ACE2 activity changes inconsistent with changes in ACE2 expression, it appeared that changes in ACE2 activity often showed a tendency similar to changes in ACE2 mRNA or protein expression. Among them, however, little research has been done on the relationship between ACE2 expression and activity and the mechanism regulating ACE2 activity. Tissue ACE2 activity is thought to be regulated by complicated mechanisms involving various factors in addition to ACE2 expression. Further investigations are awaited to address this issue.

Given that ACE2 is the receptor for SARS-CoV-2, concerns have spread that ARBs and ACEIs might be related to increased risks of SARS-CoV-2 infection and more severe COVID-19, based on the claim that some animal experiments showed overexpression of tissue ACE2 with administration of these drugs [2, 5–9]. However, this study suggested that the grounds for these concerns are quite tenuous. Moreover, the efficiency of SARS-CoV-2 infection depends on not only the amount of cell-surface ACE2 as the entrance receptor but also many other important factors including viral exposure load, binding affinity of ACE2 for the spike protein of SARS-CoV-2, internalization kinetics of the virus-ACE2 complex, and expression and activity of host cell proteases, such as transmembrane protease serine 2, that mediate spike-protein priming and facilitate cell entry following ACE2 binding [4, 107]. Indeed, an increasing number of large-scale cohort studies from China, Europe, the United States, and Japan have provided evidence that the administration of ARBs and ACEIs does not increase either susceptibility to SARS-CoV-2 infection or the risk of aggravation or mortality from COVID-19 among patients with hypertension or CVD [108–112]. Recently, a multicenter, randomized clinical trial showed that among patients who were hospitalized with mild-to-moderate COVID-19 and who were taking ACEIs or ARBs before hospital admission, there was no difference in the mean number of days alive and out of hospital for those assigned to discontinuing vs. continuing these medications [113].

In conclusion, ACE2 overexpression appeared to be rare rather than common as an effect of RASIs in the heart, kidneys, artery, lungs, intestine, and other organs in healthy animals and animal models of human diseases. Future studies should clarify the pathophysiological significance of the RASI-induced reversal or prevention of ACE2 repression in disease models. Single cell- or small cell cluster-based analyses using superselective preparation techniques would be desirable to more precisely investigate individual ACE2 expression in the epithelium/endothelium, vasculature, parenchyma, interstitium, and infiltrating cells in each organ. In terms of SARS-CoV-2 infectivity, changes in cell-surface ACE2 levels should be specifically investigated.

## Supplementary information

Supplementary Table 1

Supplementary Table 2

Supplementary Table 3

Supplementary Table 4

Supplementary Figure 1
