# Supplementary material for: Overexpression of angiotensin-converting enzyme 2 by renin-angiotensin system inhibitors. Truth or myth? A systematic review of animal studies
Source: Hypertens Res. 2021 Mar 10;44(8):955–68. doi: 10.1038/s41440-021-00641-1 (PMC7943405; doi:10.1038/s41440-021-00641-1)
Supplement: Supplementary file 1 — Supplementary Table 1 [file 41440_2021_641_MOESM1_ESM.docx]

**Supplement Table 1. Equations for the database search in MEDELINE (PubMed)**

| Step #1 | Step #2 | Step #3 | Step #4 | Step #5 |
| --- | --- | --- | --- | --- |
| ((angiotensin converting enzyme 2) OR (ACE2)) | #1 AND  (expression) | #2 AND  (animals) | #3 AND (English [Language]) | #4 AND (losartan [tiab]) |
|  |  |  |  | #4 AND (olmesartan [tiab]) |
|  |  |  |  | #4 AND (azilsartan [tiab]) |
|  |  |  |  | #4 AND (candesartan [tiab]) |
|  |  |  |  | #4 AND (telmisartan [tiab]) |
|  |  |  |  | #4 AND (valsartan [tiab]) |
|  |  |  |  | #4 AND (irbesartan [tiab]) |
|  |  |  |  | #4 AND (eprosartan [tiab]) |
|  |  |  |  | #4 AND (lisinopril [tiab]) |
|  |  |  |  | #4 AND (rampril [tiab]) |
|  |  |  |  | #4 AND (enalapril [tiab]) |
|  |  |  |  | #4 AND (captopril [tiab]) |
|  |  |  |  | #4 AND (alacepril [tiab]) |
|  |  |  |  | #4 AND (delapril) [tiab] |
|  |  |  |  | #4 AND (perindopril [tiab]) |
|  |  |  |  | #4 AND (benazepril [tiab]) |
|  |  |  |  | #4 AND (imidapril [tiab]) |
|  |  |  |  | #4 AND (temocapril [tiab]) |
|  |  |  |  | #4 AND (quinapril [tiab]) |
|  |  |  |  | #4 AND (trandolapril [tiab]) |
|  |  |  |  | #4 AND (cilazapril [tiab]) |
|  |  |  |  | #4 AND (spironolactone [tiab]) |
|  |  |  |  | #4 AND (eplerenone [tiab]) |
|  |  |  |  | #4 AND (spironolactone [tiab]) |
|  |  |  |  | #4 AND (esaxerenone [tiab]) |
|  |  |  |  | #4 AND (aliskiren [tiab]) |
|  |  |  |  | #4 AND (thiazide [tiab]) |
|  |  |  |  | #4 AND ((ARB) OR (angiotensin II receptor antagonist) OR (angiotensin receptor antagonist) OR (angiotensin receptor blocker) OR (angiotensin II antagonist) OR (AT 1 receptor blocker) OR (AT 1 receptor antagonist) OR (angiotensin receptor antagonists [Mesh]) |
|  |  |  |  | #4 AND (ACE) OR (ACE1) OR (ACEI) OR (ACE-I) OR (ACEs) OR (angiotensin-converting enzyme) OR (angiotensin-converting enzyme Inhibitors [Mesh]) |
