# Supplementary material for: Overexpression of angiotensin-converting enzyme 2 by renin-angiotensin system inhibitors. Truth or myth? A systematic review of animal studies
Source: Hypertens Res. 2021 Mar 10;44(8):955–68. doi: 10.1038/s41440-021-00641-1 (PMC7943405; doi:10.1038/s41440-021-00641-1)
Supplement: Supplementary file 5 — Supplementary Figure 1 [file 41440_2021_641_MOESM5_ESM.pptx]

## Slide 1
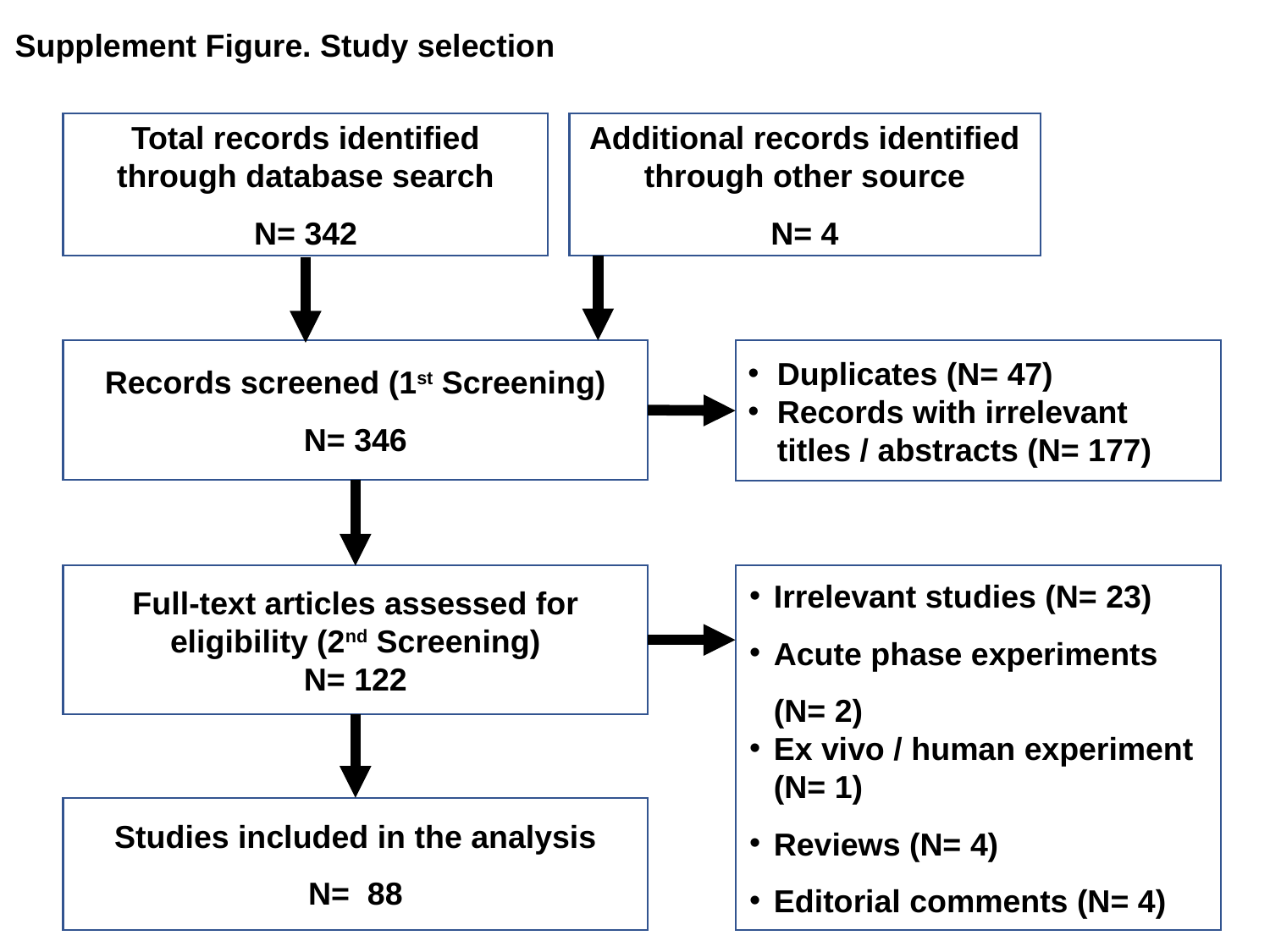

Supplement Figure. Study selection
Total records identified through database search
N= 342
Additional records identified through other source
N= 4
Records screened (1st Screening)
N= 346
Duplicates (N= 47)
Records with irrelevant titles / abstracts (N= 177)
Irrelevant studies (N= 23)
Acute phase experiments (N= 2)
Ex vivo / human experiment (N= 1)
Reviews (N= 4)
Editorial comments (N= 4)
Full-text articles assessed for eligibility (2nd Screening)
N= 122
Studies included in the analysis
N= 88
